# Middle‐aged and elderly patients with COVID‐19 pneumonia arising from asymptomatic carriers: A report of six cases

**DOI:** 10.1002/mco2.35

**Published:** 2020-11-19

**Authors:** Menglin Dai, Mulong Bao, Xian Chen, Qinxiu Zhang, Yimo Jian

**Affiliations:** ^1^ Department of Otolaryngology Hospital of Chengdu University of Traditional Chinese Medicine Chengdu People's Republic of China; ^2^ Department of Intensive Care Unit Chengdu First People's Hospital Chengdu People's Republic of China; ^3^ Department of Pathology Hospital of Chengdu University of Traditional Chinese Medicine Chengdu People's Republic of China

Dear Editor,

The entire population is susceptible during the outbreak of coronavirus disease 2019 (COVID‐19). It is difficult to diagnose patients for their different symptoms, especially the asymptomatic carrier.^1^ The real‐time reverse transcriptase‐polymerase chain reaction (RT‐PCR) swab test has become the standard for the diagnosis of COVID‐19 infection.^2^ However, four of our six clinical patients had false‐negative results in pharyngeal swab RT‐PCR tests. Among those, there was one patient, for whom four successive pharynx swab tests were negative, and only the RT‐PCR test of bronchoalveolar lavage fluid was positive.

In this report, we present the medical history, clinical manifestations, laboratory examinations, RT‐PCR tests for COVID‐19 nucleic acid, and chest computed tomography (CT) features of these patients to obtain an overall view of these asymptomatic middle‐aged and elderly patients.

Patient a who is a 47‐year‐old male, with a 1‐day history of fever and chills, was admitted to the hospital on January 31, 2020. The patient had returned from Wuhan 11 days prior and had a cough, chills, and fever 1 day previously. The highest body temperature was 38.7°C, and his hypersensitive C‐reactive protein (hs‐CRP) level was high (Figure 1C). CT showed increased lung texture, multilobular, and multisegment scattered patchy ground‐glass opacity (GGO) lesions (Figure 1A). After admission, the patient's pharynx swab tested negative on the first RT‐PCR test. On February 3, the patient tested positive by RT‐PCR (Figure 1B).

**Figure 1:**
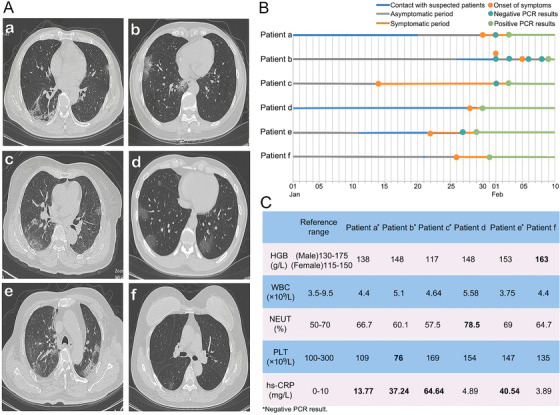
The characteristics of six patients admitted to hospital. (A) The chest CT images showed ground‐glass opacity (GGO) lesions in both lungs; (B) the asymptomatic period and RT‐PCR test results; and (C) the laboratory examinations results

Patient b who is a 49‐year‐old female, with a 5 days history of recurrent fever, was admitted to the hospital on February 6, 2020. Her husband had contact with his asymptomatic classmates in Panzhihua on January 26, 2020, and he had been diagnosed with COVID‐19 1 day prior. Additionally, the patient also had a fever 5 days prior, with the highest body temperature of 38.3°C. Subsequently, the patient was quarantined in Sichuan Gem Flower Hospital, where two RT‐PCR tests of pharyngeal swabs showed negative results. The patient visited our hospital for fever again 1 day prior. CT revealed multiple GGO lesions in the lower lobes of both lungs (Figure 1A). Besides, she had a high hs‐CRP level and a low platelet count (PLT) level (Figure 1C). Two RT‐PCR tests of pharyngeal swabs were negative after admission (February 6 and 8). Positive results were obtained only after an RT‐PCR test of bronchoalveolar lavage fluid on February 9 (Figure 1B).

Patient c who is a 78‐year‐old female, with a 16 days history of cough and expectoration, was admitted to the hospital on January 30, 2020. Three days prior, the patient also experienced fatigue, chest tightness, and shortness of breath. CT showed increased lung texture, multiple scattered patchy GGO lesions. The left pleura was thickened, adhered, and calcified (Figure 1A). Her hs‐CRP level was high (Figure 1C). After admission, the patient's RT‐PCR result was negative on the first pharynx swab test. On February 3, a second pharynx swab was positive by the RT‐PCR test (Figure 1B).

Patient d who is a 42‐year‐old male, with a 1‐day history of fever and dry throat, was admitted to the hospital on January 29, 2020. His father had died of illness 1 day prior and had developed a fever on his deathbed. His mother had recently developed a fever of unknown origin. With the highest body temperature of 37.9°C, the patient had a cough, dry throat, chest tightness, and fever one day prior. CT showed GGO lesions scattered in both lungs (Figure 1A). His neutrophil ratio (NEUT%) level was high (Figure 1C). After admission, the patient's pharynx swab tested positive on the first RT‐PCR test (Figure 1B).

Patient e who is a 65‐year‐old male, with a 5 days history of fever and chills, was admitted to the hospital on January 27, 2020. He had a history of hypertension, type 2 diabetes, and cerebral hemorrhage, and his consciousness was not clear. From January 11, 2020, the patient began to live with his asymptomatic wife who had returned from Wuhan. Five days prior, the patient had developed a fever with the highest body temperature of 38.2°C. CT suggested increased lung texture, multilobular, and multisegment scattered patchy high‐density GGO lesions (Figure 1A). His hs‐CRP level was high (Figure 1C). A pharynx swab was negative on the first RT‐PCR test after admission. On January 29, the second pharyngeal swab test was positive (Figure 1B).

Patient f who is a 55‐year‐old female, with a 5 days history of fever, was admitted to the hospital on January 31, 2020. On January 21, the patient had close contact with her asymptomatic friends. Five days prior, the patient had developed recurrent fever, with the highest body temperature of 37.6°C. CT showed patchy GGO lesions in both lungs (Figure 1A). Her hemoglobin (HGB) level was high (Figure 1C). After admission, a pharynx swab tested positive on the first RT‐PCR test (Figure 1B).

This study enrolled six middle‐aged and elderly patients with confirmed COVID‐19, with an average age of 56 (range, 42–78) years, including three females and three males. Four of the six patients had false‐negative results in pharyngeal swab RT‐PCR tests, which might be because of the weak immune response to COVID‐19 infection in the asymptomatic individuals.^3^ By contrast, the chest CT images of all the six patients showed GGO lesions in both lungs at the time of admission. Such results possibly indicated that the positive findings on chest CT appeared earlier than the positive RT‐PCR test results in asymptomatic middle‐aged and elderly patients. In addition, the average abnormal rate of their laboratory examination results was 23.33%, which was mainly associated with the high levels of hs‐CRP (13.33%). According to the above results, hs‐CRP may be an index for the auxiliary diagnosis of COVID‐19 patients. However, more clinical data are still warranted for further verification as a result of the small sample size.

Of these six patients, five had milder symptoms, while the other one with a chronic medical history had severe symptoms, which was possibly due to the severe COVID‐19 caused by his chronic diseases. By comparing their epidemiological history, it could be found that the asymptomatic COVID‐19 carriers infected four middle‐aged and elderly patients with COVID‐19 through the household transmission. It is difficult to diagnose the asymptomatic COVID‐19 carriers due to the variable or unobvious symptoms. In addition, middle‐aged and elderly patients have become the high‐risk groups for COVID‐19 infection as a result of the underlying diseases or weak self‐protection awareness and other reasons.^4^ Studies have shown that asymptomatic carriers can easily spread COVID‐19 to the middle‐aged and elderly populations through within household, which may thus cause new epidemics.^5^ As a result, for asymptomatic or suspected individuals, especially the middle‐aged and elderly people, early accurate diagnosis is essential. To improve the diagnostic accuracy of COVID‐19, the combined use of RT‐PCR and CT is recommended. In particular, in areas with insufficient conditions, CT may have a higher diagnostic value.

## CONFLICT OF INTEREST

The authors declare that they have no conflict of interest.

## ETHICS STATEMENT

The study was conducted in accordance with the principles of the Declaration of Helsinki. Our present study was approved by the Institutional Review Boards of the Chengdu First People's Hospital. The patients all agreed to publish this study via written consent. All data analyzed were anonymized.

## AUTHOR CONTRIBUTIONS

Menglin Dai and Yimo Jian designed the study. Mulong Bao collected data. Qinxiu Zhang and Xian Chen performed the analysis. Menglin Dai, Qinxiu Zhang, and Yimo Jian wrote the first draft of this paper. All authors contributed to the writing of the manuscript. All authors read and approved the final manuscript.
